# Recognition of Emotion According to the Physical Elements of the Video

**DOI:** 10.3390/s20030649

**Published:** 2020-01-24

**Authors:** Jing Zhang, Xingyu Wen, Mincheol Whang

**Affiliations:** 1 Department of Emotion Engineering, University of Sangmyung, Seoul 03016, Korea; 201934154@sangmyung.kr (J.Z.); 201934153@sangmyung.kr (X.W.); 2 Department of Human-Centered Artificial Intelligence, University of Sangmyung, Seoul 03016, Korea

**Keywords:** emotion, physical elements, emotion recognition

## Abstract

The increasing interest in the effects of emotion on cognitive, social, and neural processes creates a constant need for efficient and reliable techniques for emotion elicitation. Emotions are important in many areas, especially in advertising design and video production. The impact of emotions on the audience plays an important role. This paper analyzes the physical elements in a two-dimensional emotion map by extracting the physical elements of a video (color, light intensity, sound, etc.). We used k-nearest neighbors (K-NN), support vector machine (SVM), and multilayer perceptron (MLP) classifiers in the machine learning method to accurately predict the four dimensions that express emotions, as well as summarize the relationship between the two-dimensional emotion space and physical elements when designing and producing video.

## 1. Introduction

Emotion is an important part of our personality. Studies prove that an individual’s emotion is influenced by processes of cognition, physiological responses, motivational changes, motor activity, and subjective perception [1,2,3]. Also, information-carrying tools can result in an emotion change [4,5]. Videos, sounds, and pictures [6], as the main media currently available, have played an increasing important role in our daily lives. For example, with the increasing use of media, such as YouTube, increasingly more researchers are focusing on affective computing and sentiment analysis by extracting features, such as text, audio, and video, from these media to recognize emotions [7,8,9]. In particular, film clips or videos can give viewers more information than images or sounds [10,11,12].

The videos expose audiences to different kinds of visual and auditory sensations, which can convey narrative information and form different emotional experiences. They contain plenty of graphical, visual, and acoustic contents that can evoke people’s affective experiences, such as happiness, fear, anger, joy, and others. For a designer, it is important to focus on attracting an audiences’ attention, to entertain, and to persuade during the playing period. It is important to focus on the relationship between emotion and the feature of the video [13].

Emotions are our response to situations and are related to our being human [14]. In the related papers, the models used in videos and emotional studies can be divided into basic emotion and a circumplex model. The model of basic emotion, demonstrated by Ekman [15], contains happiness, surprise, anger, disgust, sadness, and fear, which form the base of six universal facial expressions. However, the state of people that are affected is related to and not independent of each other [16]. Thus, dimensional models of emotion have been proposed [17,18,19,20,21]. It was used by Osgood [22] and Lang [23], and finally proposed by Russell [24], which consists of two dimensions: Valence and arousal. Valence, in the horizontal axis, reflects the degree of the response from positive to negative (or “pleasant” to “unpleasant”). Arousal is the vertical axis and indicates the level of activities associated with the emotional response from very excited to very calm or sleepy. Some papers use three dimensions with another value, namely dominance, which means a feeling that controls or is controlled by the emotion [25]. However, the level of dominance is regarded as a less stable and less reliably measured dimensional response [26]; Russell [24] regards dominance as a secondary factor, and arousal and valence are primarily focused upon in emotional studies. In the present study, effective model of arousal (excited–calm) and valence (positive–negative) is commonly used, and is widely regarded as a model that is closer to representing real feelings [27]. In this study, Ekman‘s model was used for video affective content analysis.

Psychological studies have provided evidence that human emotions can be aroused by visual contents [28,29,30]. Different images can evoke different responses together with individual experiences, such as images with carnage or attractive, scantily clad people can respectively evoke emotions like disgust, horror, compassion, or sexual arousal [31]. At the same time, there are many theories used in emotional cognition that involve extracting different kinds of visual features. For example, Machajdik [32] expressed the color space using an intuitive definition of colors by defining well-separated hue (H), saturation (S), and brightness (Y) channels and segments the image to characterize its composition after resizing and cropping them; finally, 18 features in 4 groups were summarized, which are empirically proven to be related to emotional reactions. In the research of Kragel [33], a convolutional neural network was developed to decode the images and predict the valence of a human emotion rating using rich, category-specific visual features (like color intensities, spatial power spectra, and object classes). Kragel’s emotion schemas are embedded in the human visual system. In other recent work of Zhao [34], principles-of-art-based emotion features (PAEF, including balance, emphasis, harmony, variety, gradation, and movement) are extracted to classify emotions in artworks. Overall, the visual features mainly include lighting, saturation, color energy, color heat, short-change rate, shot duration, shot type transition rate, motion intensity, and motion dynamics.

Tuuri et al. [35] state that “reflexive responses represent an important way by which meanings and emotions can be evoked by sound”. Sound design can elicit affective imagery, which in turn, shapes cognition and consciousness [36]. For example, music is often considered the universal “language of emotions,” where people from a diverse range of cultures and backgrounds will often agree whether a piece of music sounds happy or sad. The audio stimuli can be music, talk, environmental sound, speech, MTV (Music Television Video), radio, etc. In Nordström’s research [37], the emotion identification points, used as the information for stable recognition, are the shortest for anger and happiness in both speech and music, but longer for music versus speech, which means that speech and music share an acoustic code for expressing emotions. The sound of an infant may be a kind of emotional response in order to overcome anxiety [38], which is also important for their future perception of emotion.

Numerous researchers have studied the extraction of audio features due to its important role in characterizing emotions. Rafael Cabredo [39] extracted 109 high-level music features using symbolic features, which were instrumentation, texture, dynamics, rhythm, pitch, statistics, and melody, and then builds a model between relaxing emotion and stressful music by using the method of linear regression. Schuller [40] used low-level descriptors (LLDs) in an open smile toolkit called AVEC and selected 30 best features from four sound groups: Cepstral (CEPS), spectral (SPEC), sound quality (SOQU: Voicing probability, logHNR, jitter, and shimmer), and prosodic (PROS: Loudness, zero-crossing rate, and F0). He concluded that arousal is highly correlated with loudness, and valence is negatively correlated with spectral flux and spectral harmonicity. Schuller also said that an independence between dimension and spectral features performs best on average, and demonstrated the feasibility of the automatic assessment of emotions in sound in two dimensions. When operating in high realism, the typical dimensional speech and music emotion recognition could be found [40]. To give a list of musical features, we can see that audio contains tempo, mode, harmony, tonality, pitch, contour, interval, rhythm, sound level, timbre, timing, articulation, accents on specific notes, tone attacks, decays, and vibrato, where different emotions are correlated with different features. [13,41]

The extraction and matching of the feature points in a video are important during the advertising production and emotion recognition [13]. The video can provide a kind of dynamic stimuli to viewers. Negative videos (usually defined as violent or horrible images) form worse memories [12] and affect the emotional information processing [42]. Positive emotions have a dominant role in consumer advertising [43] and can attract consumers’ attention from start to finish by allowing them to engage in the video advertisement [44] because such emotions induce the engagement of viewers and increases the likelihood of purchase [33]. At the same time, beautiful scenes with threatening context cues (e.g., scary music) can be reported as ominous by using frame-by-frame predictions to track meaningful variation in the film’s emotional scenes across time [45].

Videos, such as movies and MTV, contain visual and audio features that belong to five channels [46]: Visual image, print, and other graphics; music; speech; and sound or environmental effects. In the research of Mo [13], combination of Hilbert-Huang Transform(HHTC) features were extracted to cover the audio content, the visual content, and the dependencies between the audio and the visual signals, which can indicate the time-varying characteristics of visual and audio signals. These types of features can improve the recognition performance in Ekman’s emotion model. Wang [47] believes that the auditory stream (MSE: Music, speech, and environ) contains a rich source of information. In his research, audio features included an audio type proportion (ATP) and an audio scene affect vector (SAV), while visual cues were based on HLS (hue, lightness, and saturation), shot duration, visual excitement, and light key in each frame; using these, the possibility of meaningful output of basic emotions were investigated. In a music video, Zhang [48] introduced affective features by dividing arousal features and valence features, which were motion intensity, short switch rate, zero-crossing rate, tempo, and beat strength, as well as lighting, saturation, color energy, rhythm regularity, and pitch. In the analysis of MTV [49], arousal features were extracted, including motion intensity, shot switch rate, sound energy, zero-crossing rate, tempo, and beat strength, whereas the features used for valence were rhythm regularity, pitch, lighting, saturation, and color energy.

We summarize the research methods for affective computing and sentiment analysis in video media. Poria used facial expressions to analyze emotions in videos, which may less accurate with conscious expression after thinking [7]. Cambria mentioned the method of emotion calculation and analysis that used emotional words in natural language to judge and recognize emotions. This still has shortcomings because of an incomplete language knowledge base around the world [8].

In the present study, 105 video clips were used for arousal and valence subjective evaluation and 8 subjects with 6 emotion recognitions for each one. The main method of obtaining databases was to match the emotion with features of the videos through data analysis about individual emotion recognition and the emotional attribute of videos. Then, we first assumed that each feature could be used to distinguish all nine emotions and then test their ability. The result was that 18 features had a difference between each other based on ANOVA (Analysis of Variance) analysis and post hoc analysis. Finally, the obtained valued features were compared by the machine learning of three classifiers—support vector machine (SVM), k-nearest neighbors (KNN), and multilayer perceptron (MLP)—to get the best training results, which were predicted using the convolutional Neural Networks (CNN) on the dataset that we made. The contributions from this study can be summarized as follows: (1) the method of this unconscious emotional stimuli from vision and hearing was more reliable and creative than the method of recognizing faces and text recognition; (2) it was more accurate to use both statistical analysis and a deep learning MLP classifier in feature acquisition and verification; and (3) the data of significant colors, lightness, and sounds collected from typical videos were used as training data, which could then be the base for an ordinary video for emotion recognition.

## 2. Materials and Methods

### 2.1. Experiment

In this experiment, the database of videos used to recognize emotions was created. Then, eight researchers majoring in emotion recognition from an emotion engineering lab, whose combination could improve the accuracy and reliability, conducted six experiments in the method of subjective evaluation for two weeks. Each video clip contained 10-s movie scenes, which was followed by a 10-s evaluation time. The subjects were asked to watch the film clips and self-assess their emotion by reporting the felt arousal (ranging from sleepy to excited) and valence (ranging from unpleasant to pleasant) on nine-point scales. Self-assessment manikin (SAM) were shown to facilitate the self-assessments of valence (from +3 (extremely positive) to 0 (neutral) to −3 (extremely negative)) and arousal (ranging from +3 (extremely excited) to 0 (neutral) to −3(extremely calm)). A total of 105 video clips were chosen from 8 movies to create an animation. The total length of them was 40 min. All subjects gave their informed consent for inclusion before they participated in the study. The study was conducted in accordance with the Declaration of Helsinki, and the protocol was approved by the Ethics Committee of Sangmyung University, Seoul, Korea (BE2018-35).

#### 2.1.1. Modeling Method

There are nine emotions defined as the basis of Russell’s valence–arousal model: negative arousal, arousal, positive arousal, negative, neutral, positive, negative relaxed, relaxed, positive relaxed [24], found by dividing the two-dimensional space of the emotion model into nine areas.

A Likert scale with seven points was used to evaluate the emotion in terms of both arousal and valence levels and to recognize the emotional content of videos. The questionnaire style and content of a video clip is shown in Table 1. Then, the scores were mapped to the respective axis to decide the position of the emotional content of the video. The criteria for each emotion score were as follows:

1) negative arousal: valence score is −3 or −2, arousal score is +2 or +3;
2) arousal: valence score is −1.0 or +1, arousal score is +2 or +3;
3) positive arousal: valence score is +2 or +3, arousal score is +2 or +3;
4) negative: valence score is −3 or −2, arousal score is −1.0 or +1;
5) neutral: valence score is −1.0 or +1, arousal score is −1.0 or +1;
6) positive: valence score is +2 or +3, arousal score is −1.0 or +1;
7) negative relaxed: valence score is −3 or −2, arousal score is −3 or −2;
8) relaxed: valence score is +2 or +3, arousal score is −3 or −2;
9) positive relaxed: valence score is −1.0 or +1, arousal score is –3 or –2.

**Figure 1:**
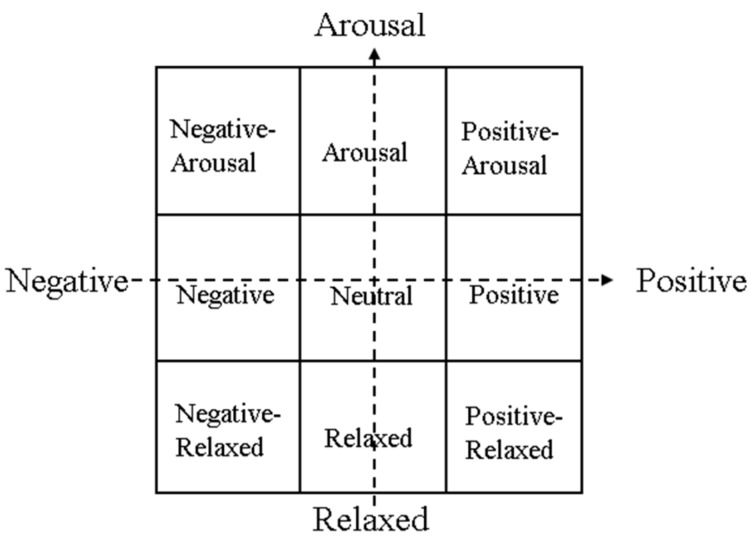
Produced valence–arousal emotion model with neutral emotions.

The model is shown in Figure 1.

#### 2.1.2. Stimuli

An important step was to establish an emotional database for the study of emotion recognition. A total of 105 film clips selected from 6 commercially animated films were shown to 8 participants. The animation mainly conveyed emotions through the changing of color, light, and sound [50]. They were more effective than ordinary videos (such as daily videos) [51]. The clip time was as short as possible to avoid creating a variety of emotions, while it was necessary to have enough time to allow for judgment.

### 2.2. Feature Extraction

**Figure 2:**
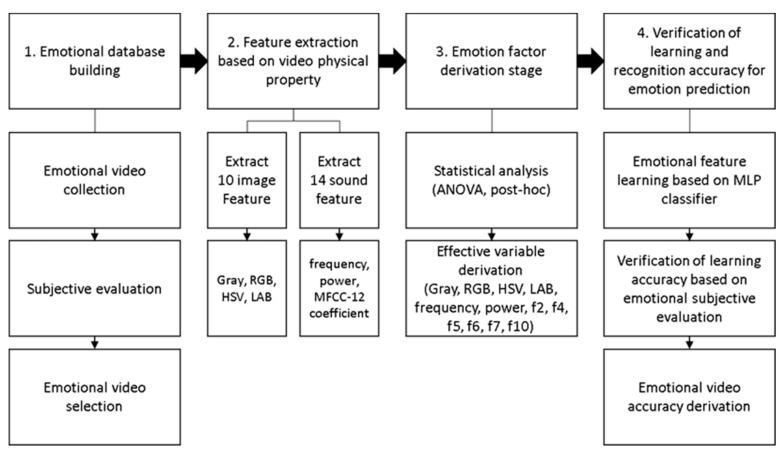
Data processing diagram. HSV: hue, saturation, value, LAB: light, the ratio of change from red to green, the ratio of change from blue to yellow, MFCC: Mel-frequency cepstral coefficients, MLP: multilayer perceptron.

There were four steps in the process of data analysis: (1) building the emotional database, (2) extracting features basing on the physical characters of the video, (3) deriving emotional factors, and (4) verifying the accuracy of the learning and predictions regarding recognizing emotions. This process is shown in Figure 2.

(1) Step one—Emotional database building.

In the step of creating the video database, these videos, representing special emotions, were collected and selected using the method of subjective evaluation. After eight professional participants had finished six experiments, it was decided that the video could be used in the database when one’s evaluation was the same over four times, and more than six participants had at least the same scores. In other words, the score of each video was the same 24 or more times. The result was that there were 78 clips in the 105 videos satisfying the above criteria that could form an emotion recognition database.

(2) Step two—Extract the features in the video.

The process of feature extraction from the videos was done in two steps. The video was mainly composed of two parts: auditory contents and visual contents, both of which evoked emotions. The features of the auditory stimuli were amplitude; a frequency, which primarily came from the video’s sound and was obtained using FFT analysis on the raw data of the sound; and 12 coefficients extracted using the method of MFCC (Mel-frequency cepstral coefficients) analysis, which was designed based on the human ear feature. The other feature was visual stimuli, which induce emotions through the changes of the chromaticity and luminosity in pictures. We extracted 30 data groups per second, which were gray, RGB (red, green, blue), HSV (hue, saturation, value), and LAB (light, the ratio of change from red to green, the ratio of change from blue to yellow).

### 2.3. Statistical Analysis

#### 2.3.1. ANOVA

To use the classifier, it needed to choose the appropriate feature between the classes. To first provide statistical analysis of the features, a series of ANOVAs were conducted to determine what features led to a difference in the valence–arousal dimensional emotions. Analysis of variance showed a significant effect of color (gray, red, green, blue) and HSV (hue, saturation, value) in the image for emotion recognition. Post hoc analyses using Tukey’s HSD were conducted by testing three a priori hypotheses by adjusting the alpha levels of 0.017 per test (0.05/3).

#### 2.3.2. Principal Component Analysis (PCA)

PCA is a method of analysis of physical features that is achieved by using advanced machine learning to select the features that can distinguish separate factors, emotions in this case. This step was undertaken in two parts: first, PCA was used as one of the methods in unsupervised machine learning, and second, these features were classified under the supervised learning methods.

#### 2.3.3. Classification Model (K-NN, SVM, MLP)

The PCA features form regular clusters that might be separated using a nonlinear hypersurface. According to the characteristics of the data, the four classification methods selected were K-NN, a nonlinear support vector machine (SVM) classifier with an adequately specified kernel function, and the deep learning MLP and CNN classifiers, which were used to classify the emotion recognition.

### 2.4. Evaluation

The data that we used to test our database in terms of the emotional recognition capabilities were from the two-dimensional emotional videos in the emotion recognition experiment. The results determined by our system were consistent with the recognized emotions of the video itself. The evaluation used CNN to divide the picture into arousal and relaxation, and then trained the data set for prediction with an accuracy rate of 90%.

## 3. Results

### 3.1. Statistical Result

The assumption we made was that the extracted physical elements could distinguish between nine emotions. The independent variables in the video were its physical elements: gray, RGB (red, green, blue), HSV (hue, saturation, value), LAB (light, ratio of change from red to green, ratio of change from blue to yellow), sound (power, frequency, MFCC-12 coefficient), which provided different emotional expressions. It was shown that the color, light, sound amplitude, and MFCC (F1–F12) in the video were the main factors influencing emotion recognition.

**Figure 3:**
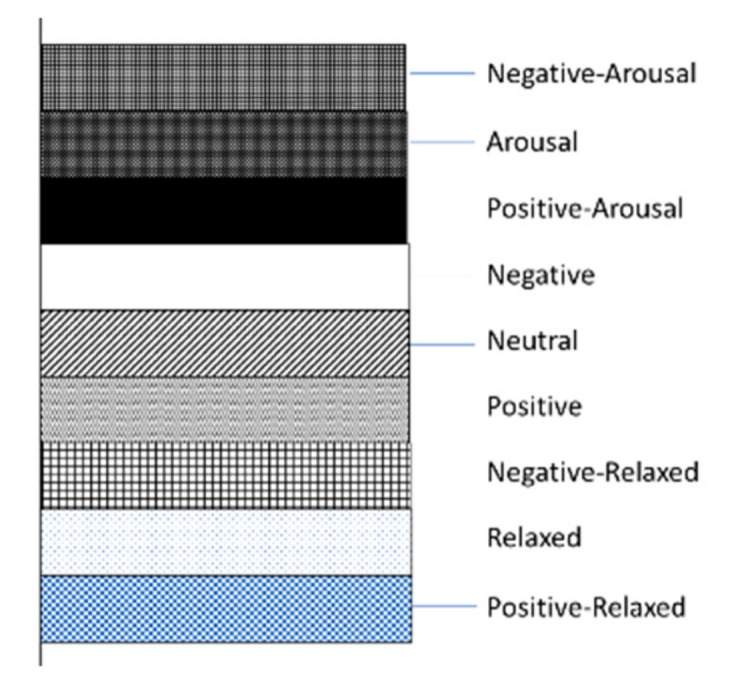
Emotional labels represented in each bar chart.

**Figure 4:**
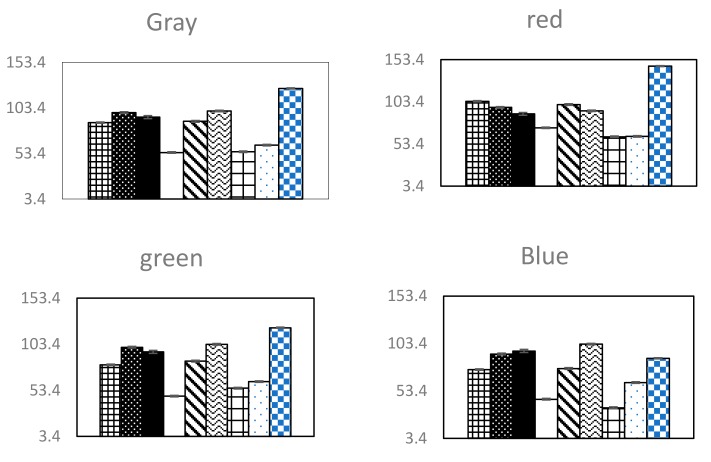

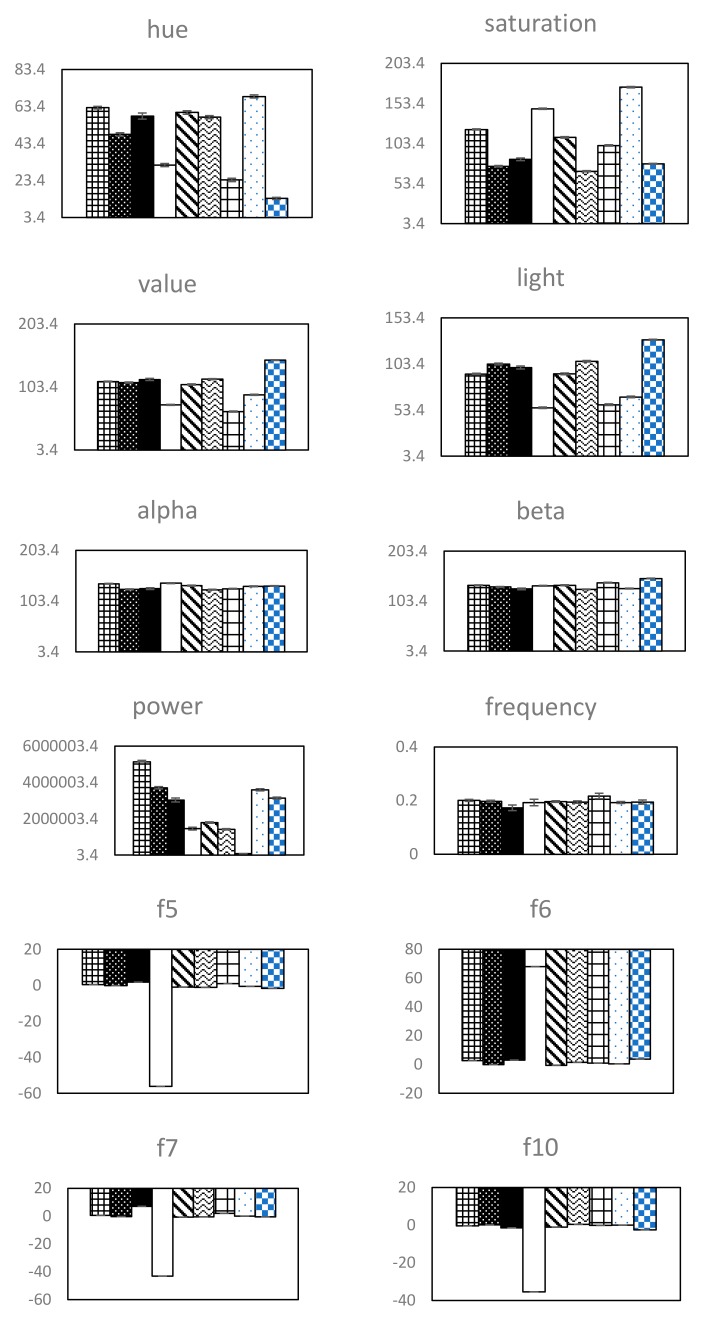
The mean error of the eigenvalues for gray, red, green, blue, hue, saturation, value, light, alpha, beta, power, frequency, f2, f4, f5, f6, f7, and f10 for the difference between each emotion comparison. The emotional order of the bars in each plot is negative arousal, arousal, positive arousal, negative, neutral, positive, negative relaxed, relaxed, and positive relaxed from left to right.

Post hoc analyses using Tukey’s HSD, which tested the three prior hypotheses, were conducted by adjusting the alpha levels of 0.017 per test (0.05/3). The results indicated that emotion recognition arousal of the three groups of participants was mainly affected by the image features (gray, red, green, blue, hue, saturation, value, light) (*p* < 0.001). The values for gray, red, green, blue, hue, saturation, value, and light of an image was the highest for arousal, but were lower for neutral and relaxed recognition in turn, e.g., the value of gray for arousal, neutral, and relaxed recognition decreased: the average number of errors was significantly lower in the group of short gray (M = 75.46, SD = 0.77; *p* = 0.001) than were those in both the group of neutral (M = 90.49, SD = 0.67; *p* = 0.001) and the group of arousal (M = 92.79, SD = 0.56; *p* = 0.001). On the contrary, the average value of saturation was higher for arousal, neutral, and relaxed recognitions: arousal (saturation M = 97.20, saturation SD = 0.68); neutral (saturation M = 100.03, saturation SD = 0.74); relaxed (saturation M = 144.60, saturation SD = 1.28). After the post analysis, the final features of identifying nine kinds of emotions were gray, red, green, blue, hue, saturation, value, light, alpha, beta, power, frequency, f2, f4, f5, f6, f7, and f10. The mean error of different characteristic data is shown in the Figure 3 and Figure 4 below.

### 3.2. Machine Learning Accuracy Result

We used Keras’ structure to train the MLP. We initialized the weights of the MLP filters using random sampling from processing with a zero mean and a 0.01 standard deviation. We trained the MLP for 100 epochs. We used a learning rate of 0.001, a weight decay of 0.0005, and a batch size of 100 samples.

**Figure 5:**
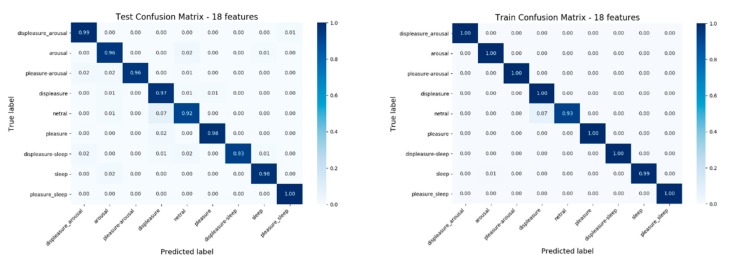
Nine emotions were used as training data and test data for the confusion matrix.

MLP requires a great number of samples to produce a model for recognizing emotion; therefore, it was necessary to increase the number of samples. For this reason, we proposed to consider randomness over the sampling method as differences lead to distinct traits. The final training accuracy result was obtained using one hundred iterative machine learning exercises was 95%. The nine emotions were used as training and test data, and are shown in the confusion matrix in Figure 5.

**Figure 6:**
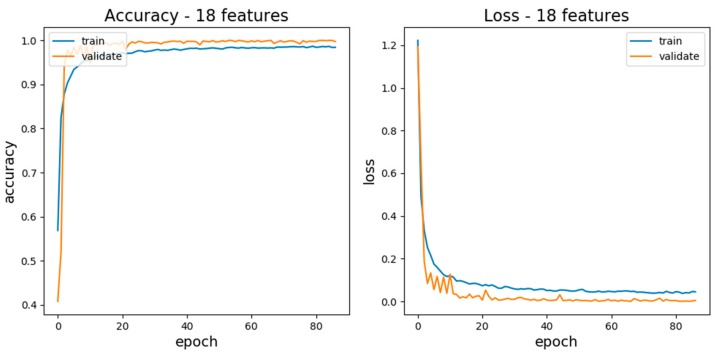
One hundred iterations through the MLP classifier, resulting in a correct rate and an error rate.

We divided the database into 10 copies which contained the training data and test data (the ratio was 7:3) using the K-NN method of cross-validation. The test accuracies of 10 copies were between 94% and 95%, as shown in Figure 6. At the same time, the test result showed that the F1 score of each emotion was high, and there was no difference in each emotion; therefore, the learning effect of the MLP model was good, as shown in Table 2.

The positive–negative dimension was predicted using a CNN, a method of machine learning that was used to classify any image in the video. The results showed that blue, black, and dark colors were always used to express negative emotion, and that other colors, such as white, yellow, and orange, were used to express positive emotion. Meanwhile, the arousal–relaxed dimension was also predicted, which showed that red, black, and dark colors were always used to express arousal, and that others, such as white, yellow, orange, and blue, expressed relaxation.

## 4. Discussion

In this paper, 105 video clips were used as the stimuli and were evaluated by eight subjects six times. The emotions used in the subjective evaluation were arousal and valence. The analysis of the subjective evaluation in this paper was based on the emotional cognition of the experiment and the two cases in which the video clip had commonality with the experimenter, and then the emotion and the features were matched in the database.

During the analysis process, we used ANOVA and PCA to extract the features that can express nine kinds of emotions. Finally, features belonging to pictures (gray, red, green, blue), and belonging to sound (hue, saturation, value, light, alpha, beta, and power extracted from power, frequency, f2, f4, f5, f6, f7, f10) were decided.

These 18 features were then classified as data sets by using three machine learning methods with high recognition performance: KNN, SVM, and MLP. By comparing the accuracy of these methods, MLP was finally decided upon as the superior method due to its accuracy of 95%. The data was randomly divided into a 7:3 ratio for the training and test data, and was classified along the arousal and valence axes using CNN for the database to provide a high classification accuracy. The result showed that arousal, relaxed, positive, and negative were four types of pictures with a higher predictive rate to be used as the picture set for comparison, and the eigenvalues were compared.

In contrast to another outcome, Poria use facial expressions to analyze emotions in videos, which may be less accurate with conscious expression after thinking [7]. In order to obtain real emotion, we used the video features such that emotions inspired by color or light were more focused upon. The audio and video were classified using positive–negative scales with a 64.86% accuracy rate obtained by the SVM machine learning classifier in Rosas research [10]. In his research, the text is one of the characteristics, which does not consider the cultural differences around the world. In our research, deep learning MLP was used to accurately classify the characteristics. Moreover, the nine categories of emotions were better than positive–negative, which can be used for future work. In conclusion, the contributions of this research are: First, elements extracted from the visual and auditory stimuli using unconscious emotional stimulus were more authentic regarding the way emotions are recognized, and the method was more innovative than facial or text recognition. Second, the extracted feature values were first subjected to statistical analysis to obtain statistically-based feature vectors, which were obtained based on the characteristics of the data set, and then deep learning MLP classifiers were used to perform feature analysis to obtain a higher classification accuracy. Third, by using color, light, and sound, animation videos with strong expression properties were selected as materials, and by extracting them, the training data was used as the emotion recognition database. In the future, emotion recognition for ordinary videos (e.g., videos of daily life) provides a good basis.

## 5. Conclusions

The conclusion is that increasing the strength of color, light, and sound in film production would enhance the viewers’ empathy in emotion recognition; this is in agreement with Suk and Hyeon, who have said that the emotional response to grays, and in particular, cool color induces more positive emotions, and more than warm colors [46]. Those colors like blue, black, and deep red are considered unpleasant, while those of white, yellow, orange, and blue are considered pleasant. Furthermore, red, black, and deep colors produce arousal, while those of white, yellow, orange, and blue produce relaxation.

In the future, we will continue to collect video clips to enrich our film clips database that can be used for different kinds of emotional recognition. Moreover, we will also find more universal features to adapt the emotion recognition.

## Figures and Tables

**Table 1:** Questionnaire style and content of a video clip.

What Kind of Emotion Do You Think is Expressed in the Video?									
Video 1		−3	−2	−1	0	1	2	3	
	Negative								Positive
	arousal								relaxed

**Table 2:** The results of the cross-validation training set.

Heading	Precision	Recall	F1 Score	Support
1	0.91	0.94	0.93	630
2	0.89	0.88	0.86	840
3	0.86	0.97	0.91	175
4	0.56	0.96	0.71	222
5	0.94	0.79	0.86	1235
6	0.92	0.96	0.94	183
7	1.00	0.94	0.97	97
8	0.93	0.96	0.94	641
9	0.99	1.00	0.99	93
